# The dual role of endoplasmic reticulum stress in cerebral ischemia: from adaptive protection to apoptotic induction

**DOI:** 10.3389/fnmol.2026.1837766

**Published:** 2026-05-07

**Authors:** Jiehong Wang, Ying Shen

**Affiliations:** Nursing Department, Sir Run Run Shaw Hospital, Zhejiang University School of Medicine, Hangzhou, China

**Keywords:** cerebral ischemia, endoplasmic reticulum stress, ER stress signaling, neuroprotection, unfolded protein response

## Abstract

Endoplasmic reticulum (ER) stress is a critical determinant of neuronal fate following cerebral ischemia, functioning as both an adaptive survival mechanism and a potent inducer of cell death. Ischemic insult and subsequent reperfusion profoundly disrupt ER proteostasis, calcium homeostasis, and redox balance, leading to activation of the unfolded protein response (UPR). While transient UPR activation promotes neuronal survival by attenuating protein synthesis, enhancing protein folding capacity, and facilitating degradation of misfolded proteins, sustained or excessive ER stress converts this adaptive response into a pro-apoptotic program that accelerates neuronal loss. This review systematically summarizes the molecular basis of ER stress in cerebral ischemia, with a particular focus on the spatiotemporal regulation of the three canonical UPR branches—PERK, IRE1α, and ATF6. We highlight how these pathways initially coordinate cytoprotective responses but subsequently drive apoptosis through CHOP induction, mitochondrial dysfunction, oxidative stress, and inflammatory signaling. The reciprocal amplification between ER stress, reactive oxygen species, and inflammatory cascades establishes a pathological network that propagates ischemic brain injury. Importantly, accumulating evidence indicates that therapeutic modulation of ER stress must be precisely timed and pathway-specific. Selectively enhancing adaptive UPR signaling while suppressing maladaptive PERK–CHOP and IRE1α–JNK pathways represents a promising strategy for neuroprotection. By integrating recent mechanistic and translational studies, this review positions ER stress as a central regulatory hub in ischemic stroke and provides a framework for the development of targeted interventions aimed at improving neurological recovery.

## Introduction

Cerebral ischemia, also known as ischemic stroke, is one of the leading causes of mortality and long-term disability worldwide, imposing a substantial burden on public health systems and affected individuals (Feigin et al., 2025; Yang et al., 2025). It results from a sudden reduction or interruption of cerebral blood flow, leading to insufficient oxygen and glucose supply to brain tissue and subsequent neuronal injury or death (Qin et al., 2022). Approximately 15 million people suffer from stroke each year globally, with an estimated 5 million dying and another 5 million suffering from long-term disabilities, including motor and cognitive impairments. Despite advances in acute reperfusion therapies, such as thrombolysis and mechanical thrombectomy, which can be effective in restoring blood flow, many patients still experience poor neurological outcomes, with about 50% of patients failing to achieve significant recovery (Ji et al., 2025; Filioglo et al., 2022). The pathological mechanisms of ischemic injury are multifactorial, involving inflammation, oxidative stress, mitochondrial dysfunction, and activation of apoptotic pathways. Therefore, a deeper understanding of the cellular and molecular mechanisms underlying cerebral ischemia, including the role of endoplasmic reticulum stress and its interaction with inflammation and oxidative stress, is essential for the development of more effective neuroprotective strategies and adjunctive treatments (Meng et al., 2025; Yang et al., 2025; Kumar Saini and Singh, 2024).

Accumulating evidence indicates that endoplasmic reticulum stress is a critical pathological event in cerebral ischemia and plays an important role in determining neuronal fate after ischemic injury (Han et al., 2021; Kang et al., 2024; Zheng et al., 2025). Cerebral ischemia leads to severe disturbances in cellular homeostasis, including energy depletion, calcium dysregulation, oxidative stress, and accumulation of misfolded or unfolded proteins within the ER lumen, thereby triggering ER stress. In response, cells activate the unfolded protein response to restore ER function and promote survival (Zhang et al., 2022). However, when ischemia is prolonged or reperfusion injury exacerbates cellular damage, excessive or sustained ER stress can overwhelm adaptive mechanisms and initiate apoptotic signaling pathways (Merighi and Lossi, 2022). Thus, ER stress serves as an important molecular link between ischemic insult and neuronal injury. The context-dependent consequences of ER stress signaling during cerebral ischemia will be discussed in detail in the following sections.

Endoplasmic reticulum stress is a cellular response triggered by an imbalance between the demand for protein folding and the protein folding capacity of the ER (Bhattarai et al., 2021; Xu and Wang, 2024). The ER, a multifunctional organelle responsible for protein synthesis, folding, and post-translational modifications, is highly sensitive to changes in cellular homeostasis (Schwarz and Blower, 2016). Under normal conditions, the ER can efficiently process proteins and assist in their proper folding. However, when cells encounter stressors such as nutrient deprivation, oxidative stress, calcium imbalance, or hypoxia, these processes can be disrupted, leading to the accumulation of unfolded or misfolded proteins within the ER (Celik et al., 2023). In response to this, the cell activates the unfolded protein response, a protective mechanism aimed at restoring protein homeostasis (Villalobos-Labra et al., 2019). The UPR is mediated by three main ER-resident sensors: protein kinase RNA-like ER kinase (PERK), inositol-requiring enzyme 1 (IRE1), and activating transcription factor 6 (ATF6), each of which regulates specific downstream pathways that control protein folding, degradation, and cellular survival (Frakes and Dillin, 2017). When ER stress is mild or transient, the UPR helps the cell adapt by reducing protein load and enhancing protein-folding capacity, promoting survival (Jin et al., 2025; Wang et al., 2018). However, if stress is prolonged or overwhelming, the UPR can shift from a protective to a pathological response, leading to apoptosis or necrosis (Hetz and Papa, 2018).

In the context of cerebral ischemia, ER stress plays a pivotal role in neuronal injury. Cerebral ischemia, resulting from an interruption of blood flow to the brain, causes severe metabolic disturbances, including oxygen and glucose deprivation (Badiola et al., 2011; Benavides et al., 2005). These conditions create a cellular environment that exacerbates protein misfolding and induces ER stress (Zhou et al., 2024). Furthermore, reperfusion after ischemic injury can amplify ER stress through the generation of reactive oxygen species (ROS), calcium overload, and inflammatory responses (Liao et al., 2026; Yin et al., 2025). As a result, neurons exposed to ischemic stress experience a compromised ability to fold proteins properly, triggering the activation of the UPR (Lei et al., 2018). In an attempt to restore normal ER function, this response can initially offer neuroprotection, but if ischemia persists or is followed by reperfusion injury, the UPR becomes maladaptive, leading to neuronal apoptosis (Lei et al., 2018; Xu et al., 2021). This balance between adaptive and maladaptive ER stress responses is crucial in determining the extent of neuronal damage in cerebral ischemia.

The involvement of endoplasmic reticulum stress in cerebral ischemia highlights its unique importance in stroke pathophysiology and its potential as a therapeutic target (Thangaraj et al., 2020). While mild ER stress can promote cellular adaptation and survival, excessive or sustained ER stress significantly contributes to neuronal injury and death in ischemic stroke. Given that ER stress is a central mediator of ischemic damage, modulating the unfolded protein response pathways offers a promising strategy to reduce brain injury and improve recovery in stroke patients (Sun et al., 2024). Pharmacological agents aimed at enhancing the adaptive phase of the UPR or preventing the shift to pro-apoptotic signaling could serve as neuroprotective therapies. Moreover, targeting specific components of the UPR, such as PERK inhibitors or chemical chaperones that assist in protein folding, has shown potential in preclinical models of ischemic stroke (Kubota et al., 2006; Komoike and Matsuoka, 2016). In addition, there is a complex interaction between ER stress, inflammation, mitochondrial dysfunction, and oxidative stress (especially the excessive production of reactive oxygen species, ROS) after cerebral ischemia. These pathological processes amplify one another, further exacerbating neuronal injury (Kovacheva et al., 2025; Yuan et al., 2025). For instance, ROS not only directly damage protein structures but also activate key signaling pathways, such as IRE1α/TRAF2/ASK1, which promote the shift from adaptive ER stress responses to pro-apoptotic signaling (Zhang et al., 2022). This interplay between ROS and ER stress enhances the neurotoxic effects of ischemic injury, making it critical to understand how these pathways cross-talk (Nakka et al., 2016). By modulating ER stress and the associated inflammatory and oxidative responses, we may be able to reduce neuronal damage and improve clinical outcomes for stroke patients.

Therefore, the aim of this review is not only to summarize the current knowledge regarding endoplasmic reticulum stress in cerebral ischemia, but also to address several conceptual gaps that remain insufficiently clarified in the existing literature. Although previous studies have described the “dual role” of ER stress in ischemic injury, the mechanisms underlying the transition from adaptive protection to apoptotic induction remain incompletely understood. In particular, the temporal regulation of unfolded protein response signaling, the crosstalk between ER stress and other pathological processes such as oxidative stress, mitochondrial dysfunction, and neuroinflammation, and the therapeutic implications of selectively modulating specific UPR branches have not been systematically integrated.

In this review, we provide a comprehensive framework that places ER stress within a dynamic spatiotemporal regulatory network during cerebral ischemia. We highlight how the three canonical UPR branches—PERK, IRE1α, and ATF6—exert stage-dependent effects on neuronal survival and death, and how their interactions with mitochondrial signaling, reactive oxygen species, and inflammatory cascades shape ischemic outcomes. Furthermore, we discuss emerging translational strategies aimed at precisely modulating ER stress responses, emphasizing the importance of timing and pathway specificity for neuroprotection. By integrating recent mechanistic and translational studies, this review offers a refined perspective on the context-dependent role of ER stress and provides theoretical support for the development of targeted therapeutic interventions in cerebral ischemia.

## Endoplasmic reticulum stress (ERS)

Endoplasmic reticulum stress refers to a highly conserved defensive response triggered by the accumulation of misfolded or unfolded proteins in the endoplasmic reticulum that exceeds the ER’s folding and processing capacity (Bravo et al., 2013; Zhang et al., 2005). Biologically, ERS is not a singular pathological event but rather a self-regulation and fate decision mechanism initiated by cells when protein homeostasis (proteostasis) is impaired. It serves as a core junction between protein quality control and the decision of whether the cell will survive or undergo cell death (Tsang et al., 2010). Functionally, the ER can be viewed as the “protein quality control and processing center” within the cell, responsible for folding, modification, and transport of newly synthesized proteins (Braakman and Hebert, 2013). When this “quality control center” is overloaded due to insufficient capacity or an excess of defective proteins, ER stress is activated, indicating the cell is at risk of functional imbalance.

ERS is not triggered by a single factor, but rather results from a combination of various internal and external disturbances. Any factor that disrupts the protein folding environment or increases the protein folding load can induce ERS, with the underlying essence being the imbalance between “workload” and “processing capacity” (Cao and Kaufman, 2014; Vietri et al., 2025). First, energy and nutritional imbalances are major causes of ERS. Under conditions such as hypoxia, hypoglycemia, or ischemia, ATP production is limited, and the energy-dependent folding processes in the ER are suppressed. Additionally, metabolic abnormalities, such as acidosis, can interfere with protein structural stability, promoting the accumulation of misfolded proteins. Second, disturbances in redox balance are also key triggers (Oliveira et al., 2026; Chai et al., 2025). The oxidative folding of proteins in the ER is highly sensitive to the redox environment, and under pathological conditions, the generation of reactive oxygen species disrupts this fine balance, directly damaging folding proteins and amplifying the stress response (Wang and Wang, 2023; Jha et al., 2021). Third, calcium homeostasis disruption plays a central role in ERS. The ER is the main intracellular calcium storage site, and calcium ions are involved in signal transduction and the normal function of many molecular chaperones and folding enzymes (Pontisso et al., 2024; Carreras-Sureda et al., 2023; Jiang et al., 2021). Any factor that causes abnormal calcium loss from the ER, such as toxins, metabolic products, or membrane permeability changes, can significantly weaken protein folding capacity and trigger stress. Furthermore, excessive protein synthesis load is also an important inducer, especially in cells with high secretory or metabolic activity, such as neurons, pancreatic *β*-cells, and hepatocytes (Carreras-Sureda et al., 2023; Mbara et al., 2024). In these cells, protein synthesis demands remain high under normal conditions, and when environmental conditions worsen, folding pressure increases rapidly. Finally, exogenous factors such as certain drugs, toxins, and viral infections can directly interfere with ER function or hijack the host protein synthesis system, triggering ERS. Overall, all these factors ultimately lead to one common outcome: the protein folding demand exceeds the ER’s processing capacity, initiating ER stress.

**Figure 1:**
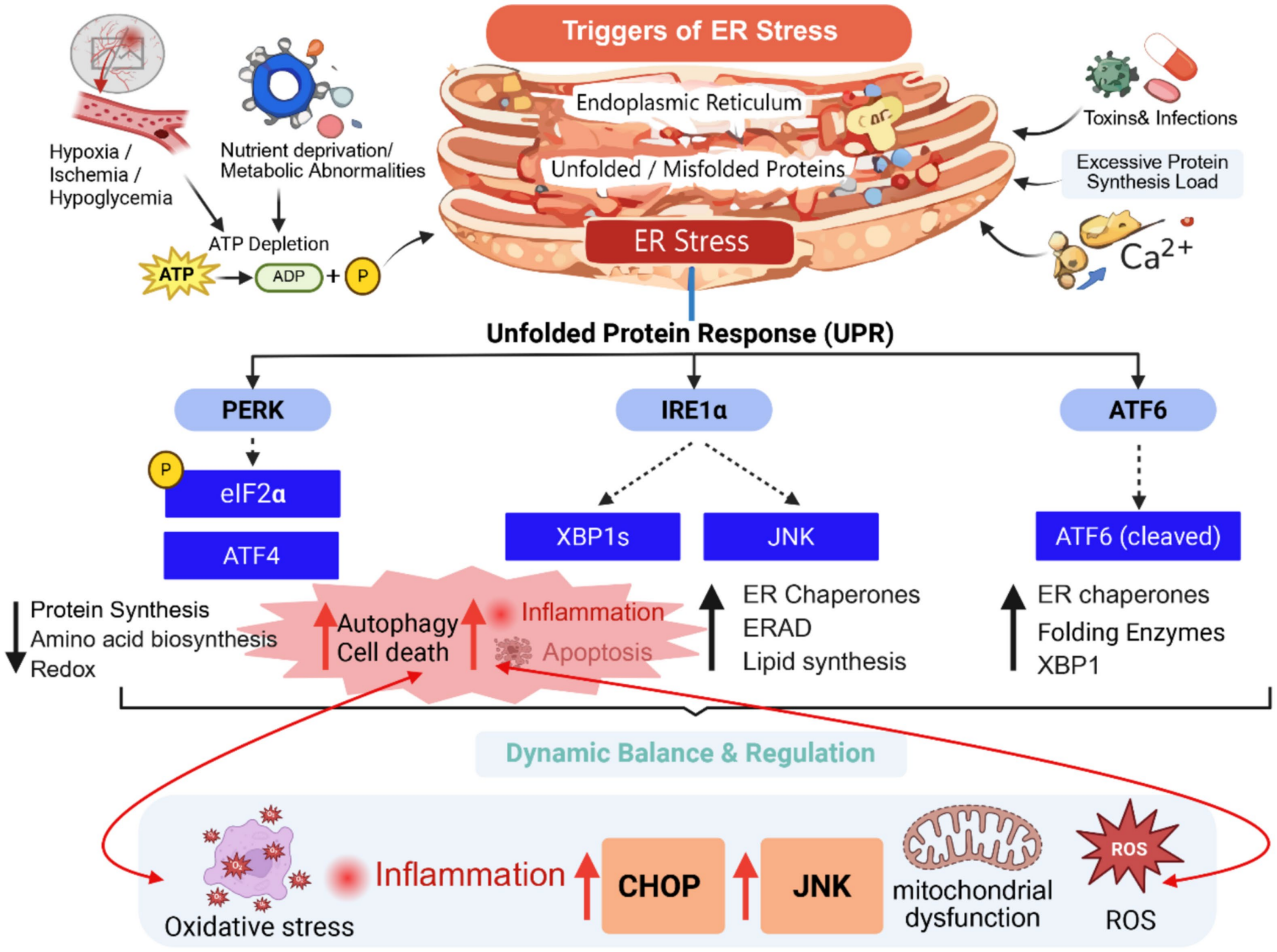
The dual role of endoplasmic reticulum stress in cerebral ischemia.

To cope with endoplasmic reticulum stress, cells have evolved a highly conserved and sophisticated signaling network known as the unfolded protein response (Ong et al., 2024; Matsushima et al., 2025). These molecular mechanisms have been extensively characterized in diverse biological systems, including cancer, metabolic diseases, and cardiovascular disorders. However, many of these mechanistic insights originate from non-neuronal models. Evidence directly derived from cerebral ischemia models, including *in vivo* stroke models and *in vitro* oxygen–glucose deprivation experiments, has gradually emerged in recent years. The primary goal of the UPR is to restore ER homeostasis, and its core strategies can be summarized as reducing the workload, enhancing the processing capacity, and clearing misfolded proteins (Kim, 2024). The UPR is mediated by three key sensors located on the ER membrane: IRE1α, PERK, and ATF6. These sensors regulate cellular stress responses through distinct yet cooperative signaling pathways (Ong et al., 2024). The IRE1α pathway is the oldest and most conserved branch of the UPR. IRE1α possesses both kinase and ribonuclease activities (Joshi et al., 2025). Upon activation during ER stress, IRE1α dimerizes and autophosphorylates, specifically cleaving X-box binding protein 1 (XBP1) mRNA to generate the transcriptionally active XBP1s. XBP1s then enters the nucleus, upregulating genes involved in protein folding, secretion pathway expansion, and ER-associated degradation (ERAD), performing primarily adaptive and pro-survival functions (Silva et al., 2025). In the context of cerebral ischemia, activation of the IRE1α–XBP1 signaling axis has also been observed in experimental stroke models and OGD-treated neurons, suggesting that this pathway contributes to adaptive responses and inflammatory regulation during ischemic injury. The PERK pathway acts as a rapid response mechanism during stress. Upon activation, PERK phosphorylates the translation initiation factor eIF2α, rapidly inhibiting global protein translation and significantly reducing the ER load (Le et al., 2025). Simultaneously, this pathway allows for the selective translation of ATF4, which further regulates antioxidant responses, amino acid metabolism, autophagy, and apoptosis-related genes, giving the PERK pathway dual roles in cell adaptation and pro-apoptotic effects (Liu et al., 2024). Several studies have further confirmed activation of the PERK–eIF2α–ATF4–CHOP signaling pathway in cerebral ischemia and oxygen–glucose deprivation models, where excessive activation contributes to neuronal apoptosis and ischemic brain injury. The ATF6 pathway primarily functions through transcriptional regulation. Upon ER stress, ATF6 translocates from the ER to the Golgi, where it is cleaved by proteases to release the active form, ATF6(N) (Oka et al., 2022). ATF6(N) enters the nucleus, where it collaborates with XBP1s to upregulate molecular chaperones and folding enzymes, enhancing the ER’s processing capacity and mediating adaptive protective responses (Vidal et al., 2021). Evidence from experimental models of cerebral ischemia further suggests that ATF6 activation may exert neuroprotective effects during the early stages of ischemic stress by enhancing adaptive ER stress responses. It is important to note that ER stress does not occur in isolation but is closely linked to mitochondrial dysfunction, oxidative stress, and inflammatory responses. In ischemic brain injury, for example, the accumulation of ROS further damages ER function, while ER stress, through pathways such as CHOP, can exacerbate ROS generation, creating a vicious cycle of cellular damage. Moreover, metabolic regulators like SIRT1 play a significant role in modulating the UPR by deacetylating key proteins involved in ER stress regulation, such as ATF6 and XBP1, thereby influencing the adaptive response (Zhao et al., 2024; Chen et al., 2023). The IRE1α/TRAF2/ASK1 pathway acts as a bridge between ER stress, inflammation, and apoptosis, with its activation promoting JNK phosphorylation, further amplifying stress-induced damage and neuronal death (Kang et al., 2024). Thus, the ER stress/UPR system functions as a dynamically regulated signaling network whose outcomes depend on the intensity, duration, and cellular context of the stress. Importantly, accumulating evidence from cerebral ischemia models indicates that the balance between adaptive and pro-apoptotic UPR signaling plays a crucial role in determining neuronal survival and functional recovery after stroke. The associated diagram (Figure 1) summarizes the key molecular pathways involved in this complex network, illustrating the dual role and dynamic balance between adaptive and pro-apoptotic responses in cerebral ischemia.

This diagram summarizes the key molecular pathways involved in the complex network of endoplasmic reticulum stress during cerebral ischemia. It illustrates the dynamic balance between adaptive and pro-apoptotic responses. In the early stages of ischemia, the unfolded protein response pathways, including PERK, IRE1α, and ATF6, initiate adaptive mechanisms to protect neurons, such as enhancing protein folding capacity and reducing protein synthesis. However, prolonged or severe ER stress leads to the activation of pro-apoptotic pathways, particularly through the PERK-ATF4-CHOP axis and the IRE1α-TRAF2-ASK1-JNK cascade, contributing to neuronal death. The diagram emphasizes the transition from protective to destructive responses as a result of the intensity and duration of the stress.

## Cell-type-specific ER stress responses in cerebral ischemia

Cerebral ischemic injury is not solely a neuron-centered process but rather a complex pathological event involving multiple cellular components of the neurovascular unit, including astrocytes, microglia, endothelial cells, and oligodendrocytes (Qin et al., 2022; Han et al., 2021). Increasing evidence suggests that endoplasmic reticulum stress responses exhibit substantial cell-type specificity, which critically influences the progression of ischemic brain injury (Zheng et al., 2025; Nakka et al., 2016).

In neurons, excessive ER stress is primarily associated with activation of pro-apoptotic pathways. Prolonged activation of PERK–eIF2α–ATF4 signaling promotes CHOP expression, leading to mitochondrial dysfunction, oxidative stress, and ultimately neuronal apoptosis (Merighi and Lossi, 2022). In contrast, moderate activation of the unfolded protein response can transiently enhance neuronal survival by restoring protein folding homeostasis and reducing misfolded protein accumulation (Han et al., 2021).

Astrocytes play a crucial neuroprotective role during ischemic injury. ER stress signaling in astrocytes has been shown to regulate inflammatory responses, glutamate uptake, and metabolic support for neurons. Activation of adaptive UPR pathways may enhance astrocytic resilience and promote neuronal protection. However, persistent ER stress can also trigger inflammatory signaling cascades that exacerbate secondary brain injury (Nakka et al., 2016).

Microglia represent the primary immune cells of the central nervous system. ER stress in microglia is closely linked to neuroinflammation. Activation of the IRE1α and PERK pathways can promote the production of pro-inflammatory cytokines through downstream signaling pathways such as NF-κB activation and NLRP3 inflammasome signaling, thereby amplifying inflammatory responses following ischemia (Han et al., 2021; Nakka et al., 2016). Conversely, controlled ER stress responses may contribute to the resolution of inflammation and tissue repair (Zheng et al., 2025).

Endothelial cells and oligodendrocytes are also highly sensitive to ER stress during ischemic conditions. In endothelial cells, ER stress contributes to blood–brain barrier disruption by regulating tight junction proteins such as ZO-1, occludin, and claudin-5, thereby increasing vascular permeability during ischemic injury (Qin et al., 2022; Zheng et al., 2025). Oligodendrocytes, which are responsible for myelin maintenance, are particularly vulnerable to ER stress–induced apoptosis, leading to white matter injury after ischemic stroke (Merighi and Lossi, 2022).

Taken together, these findings indicate that ER stress responses are highly context-dependent and vary significantly among different cell types within the neurovascular unit (Han et al., 2021; Zheng et al., 2025). Understanding these cell-specific mechanisms may provide important insights for developing targeted therapeutic strategies aimed at modulating ER stress in cerebral ischemia (Xu and Wang, 2024). A summary of the major ER stress pathways and pathological outcomes in different cell types is provided in Table 1.

**Table 1:** Cell-type-specific ER stress responses in cerebral ischemia.

Cell type	Major ER stress pathways	Main pathological effects	Potential outcomes
Neurons	PERK–eIF2α–ATF4–CHOP	Apoptosis, oxidative stress	Neuronal death
Astrocytes	PERK, ATF6	Regulation of inflammation, metabolic support	Neuroprotection or inflammation
Microglia	IRE1α, PERK	Cytokine production, inflammatory amplification	Neuroinflammation
Endothelial cells	PERK, ATF6	Blood–brain barrier disruption	Vascular dysfunction
Oligodendrocytes	PERK–CHOP	ER stress–induced apoptosis	Demyelination

ER, endoplasmic reticulum; UPR, unfolded protein response; PERK, protein kinase RNA-like ER kinase; IRE1α, inositol-requiring enzyme 1α; ATF6, activating transcription factor 6.

## The adaptive role of endoplasmic reticulum stress in cerebral ischemia

In the early stages of cerebral ischemia or in cases of mild ischemia, endoplasmic reticulum stress typically manifests as an adaptive protective response (Han et al., 2021). The energy depletion and protein folding disturbances induced by ischemia and hypoxia can quickly activate ER stress signaling, prompting cells to initiate multi-layered defense mechanisms to maintain ER function and overall cellular homeostasis (Zheng et al., 2025). On one hand, by activating the unfolded protein response, cells temporarily suppress the synthesis of new proteins, thereby reducing the burden on the ER. Simultaneously, they upregulate the expression of molecular chaperones and folding-related enzymes, enhancing their ability to process misfolded proteins. On the other hand, the UPR also accelerates the clearance of misfolded proteins and damaged organelles through ER-associated degradation (ERAD) and autophagy, preventing the aggregation of toxic proteins and secondary damage caused by these aggregates (Hwang and Qi, 2018). In the pathological context of cerebral ischemia, this adaptive ER stress response aimed at restoring homeostasis helps increase neuronal tolerance to ischemic stress, delays the progression of cell injury, and buys valuable time for subsequent reperfusion repair and neurological recovery (Marciniak et al., 2022).

The three classic unfolded protein response signaling pathways are not activated simultaneously or equally in cerebral ischemia, but exhibit distinct temporal sequences and functional differences, all contributing to neuroprotection. After ischemia occurs, the PERK pathway is typically activated first. By phosphorylating eIF2α, it rapidly inhibits global protein translation, effectively alleviating the ER load. Simultaneously, the PERK–eIF2α–ATF4 axis induces the expression of various autophagy-related genes (B'chir et al., 2013; Le et al., 2025). This moderately enhanced autophagy helps clear damaged organelles and misfolded proteins, playing an important role in cellular protection during the early stages of cerebral ischemia. The IRE1α-mediated XBP1s signaling pathway primarily helps restore ER homeostasis and regulates the expression of inflammation-related genes, limiting the excessive release of inflammatory factors and reducing secondary inflammatory damage in the ischemic region (Dong et al., 2025; Zhang et al., 2022). In contrast, the ATF6 pathway mainly enhances the ER’s protein processing capacity by upregulating the expression of molecular chaperones (such as GRP78/BiP) and various folding-related enzymes. In several cerebral ischemia models, the activation of ATF6 is considered a typical adaptive protective signal, and its enhancement helps alleviate the extent of ER stress and improves neuronal survival rates (Yu et al., 2025). During the adaptive ER stress phase, several protective pathways, such as the ERK5/MEF2A pathway, also play crucial roles by promoting the expression of anti-apoptotic genes, inhibiting inflammation, and enhancing neuronal tolerance to ischemic stress (Yin et al., 2021). Pharmacologically, activation of ERK5 signaling has been explored using agents such as dexmedetomidine and netrin-1, which have been reported to attenuate ER stress and improve neurological outcomes in experimental models of cerebral ischemia. These findings suggest that modulation of ERK5/MEF2A signaling may represent a promising strategy for neuroprotective intervention. Additionally, ER stress regulatory proteins such as SERP1 are involved in maintaining protein folding homeostasis. These mechanisms work together to ensure cell survival under mild ischemia or in oxygen–glucose deprivation (OGD) models (Yin et al., 2025). These coordinated responses help neurons cope with the stress of ischemia and limit the extent of damage in the early stages of the ischemic event.

## Mechanisms of endoplasmic reticulum stress-induced apoptosis

As the duration of ischemia increases or reperfusion injury occurs, the local microenvironment in cerebral ischemia rapidly deteriorates, with persistent hypoxia, ischemia, energy depletion, oxidative stress, and calcium homeostasis disruption compounding one another (Lan et al., 2024). These factors lead to long-term damage to the endoplasmic reticulum, causing ER function to deteriorate, and ER stress gradually shifts from an initial adaptive response to a pathological one. In this context, the unfolded protein response is no longer effective in restoring ER homeostasis but instead continuously activates downstream pro-apoptotic signaling pathways, becoming a crucial mechanism driving neuronal death (Zheng et al., 2025). When ER stress becomes too intense or persists for too long, the UPR signals shift from survival to apoptosis, fully demonstrating the “double-edged sword” effect of ER stress.

Mechanistically, the sustained activation of IRE1α can recruit TRAF2 and further activate the ASK1–JNK pathway, amplifying stress-induced damage signals. At the same time, long-term activation of the PERK–ATF4 pathway significantly upregulates various pro-apoptotic genes, among which C/EBP homologous protein (CHOP) is considered a key transcription factor mediating apoptosis induced by ER stress (Kang et al., 2024). CHOP is significantly upregulated in cerebral ischemia and amplifies apoptotic signals by inhibiting anti-apoptotic proteins like Bcl-2, enhancing the generation of reactive oxygen species, and disrupting cellular metabolism (Wu et al., 2022). Additionally, CHOP and related signaling pathways promote the expression of pro-apoptotic proteins like Bax, triggering changes in mitochondrial permeability and activating the caspase cascade, ultimately leading to neuronal programmed cell death (Rabachini et al., 2018). As a core transcription factor mediating apoptosis induced by ER stress, CHOP not only suppresses anti-apoptotic proteins such as Bcl-2 but also promotes the expression of pro-apoptotic proteins like Bax, further activating the caspase cascade (Chen et al., 2024). Moreover, CHOP enhances ROS generation, exacerbating oxidative stress and mitochondrial dysfunction. In models such as oxygen–glucose deprivation, the high expression of CHOP correlates significantly with the extent of neuronal apoptosis. The upregulation of CHOP, combined with these effects, shifts cellular responses toward apoptosis, contributing to neuronal death through the activation of caspases and mitochondrial permeabilization (Wang et al., 2024). Furthermore, the RhoA/Pyrin pathway plays an important role in linking ER stress with inflammasome activation, amplifying the inflammatory damage after ischemia (Fang et al., 2025). From a therapeutic perspective, pharmacological modulation of the RhoA signaling axis, including the use of Rho kinase (ROCK) inhibitors, has been investigated in ischemic stroke models and has shown potential to reduce neuroinflammation and improve cerebral blood flow. These findings suggest that targeting the RhoA/Pyrin signaling pathway may provide a feasible strategy for mitigating ER stress–associated inflammatory injury. Thus, the interplay between ER stress, CHOP-induced apoptosis, oxidative stress, mitochondrial dysfunction, and inflammation through the RhoA/Pyrin axis underscores the complex mechanisms that drive neuronal death in ischemic stroke. The degree of neuronal apoptosis is closely correlated with the extent of cerebral ischemia and the degree of neurological dysfunction. ER stress-induced apoptosis not only directly reduces the number of surviving neurons but also exacerbates inflammation and blood–brain barrier disruption, further expanding the brain damage area and significantly affecting patient prognosis.

## The dual role of endoplasmic reticulum stress in cerebral ischemia

Extensive basic research and animal experiments have demonstrated that endoplasmic reticulum stress plays a dual role in cerebral ischemia, exhibiting both adaptive protective effects and apoptotic induction, which together present a significant dual effect (Chen et al., 2025). During the early stages of ischemia or in stages where stress is still reversible, the unfolded protein response primarily exerts neuroprotective effects by restoring ER homeostasis and enhancing protein folding and degradation capacity. However, in the stage of prolonged ischemia or reperfusion injury, UPR signaling gradually shifts toward pro-apoptotic pathways, accelerating neuronal death. Studies from clinical samples and animal models further indicate that the expression levels of ERS-related molecules (such as GRP78 and CHOP) are closely correlated with the stage of cerebral ischemia and the severity of the condition, providing strong evidence for the spatiotemporal dependence of ERS (Zha et al., 2025).

Therefore, ERS is not simply a matter of “inhibition or activation,” but the key lies in maintaining a dynamic balance: excessive inhibition of ERS may weaken the cell’s ability to adapt, while prolonged or excessive activation accelerates cell apoptosis. Thus, precisely regulating the activation strength and duration of UPR signaling pathways is considered a core strategy for achieving effective neuroprotection. Based on this understanding, targeting ER stress and the UPR pathways has become an important potential therapeutic direction for neuroprotection after cerebral ischemia. Current research mainly focuses on the selective regulation of the PERK, IRE1, and ATF6 pathways, where inhibiting the excessive activation of PERK or IRE1 signaling helps reduce apoptotic responses, while enhancing the activity of ATF6 or XBP1s may strengthen the adaptive protective effects. In addition, chemical chaperones, antioxidants, and other drugs that improve the protein folding environment or alleviate oxidative stress have also shown promising neuroprotective potential in various cerebral ischemia models (Bosten et al., 2022).

However, despite the positive progress in experimental studies, the clinical translation of these strategies still faces challenges, such as narrow therapeutic windows, differences in responses between cell types, and potential systemic side effects (Bravo et al., 2013). Future research needs to focus on refining and personalizing the regulation of endoplasmic reticulum stress to promote the establishment of precise treatment strategies for cerebral ischemia. Future therapeutic approaches targeting ERS should take into account its intersection and cross-regulation with pathways related to inflammation, mitochondrial function, and oxidative stress (Nakka et al., 2016). For example, modulating SIRT1 activity may simultaneously improve both ERS and metabolic states, while inhibiting the IRE1α/TRAF2/ASK1 pathway could alleviate inflammation and apoptosis (Qing et al., 2023). Enhancing the ERK5/MEF2A signaling pathway might offer neuroprotection by promoting anti-apoptotic gene expression and reducing oxidative stress (Yang et al., 2025; Xiao et al., 2023; Wang et al., 2025). Moreover, regulatory proteins like SERP1, identified in the OGD model, could also serve as novel therapeutic targets, providing further avenues for precise and effective interventions.

## Conclusion and perspectives

In summary, endoplasmic reticulum stress plays a complex and context-dependent role in the pathogenesis and progression of cerebral ischemia. Accumulating evidence indicates that moderate activation of the unfolded protein response may promote adaptive cellular responses and neuronal survival, whereas excessive or prolonged ER stress can trigger apoptotic signaling pathways and aggravate neuronal injury. These dual effects highlight the importance of precisely regulating ER stress signaling during ischemic brain injury.

Despite substantial progress in elucidating the molecular mechanisms of ER stress in cerebral ischemia, several unresolved issues remain. In particular, the temporal dynamics of ER stress activation during different stages of ischemia and reperfusion are still incompletely understood. Moreover, the cell-type–specific roles of ER stress within the neurovascular unit, including neurons, astrocytes, microglia, endothelial cells, and oligodendrocytes, require further investigation to clarify how ER stress contributes to both neuroprotection and neurodegeneration.

There are also points of contention regarding whether ER stress predominantly acts as a protective adaptive response or a driver of neuronal injury in different pathological contexts. These discrepancies may arise from variations in experimental models, differences in the timing and intensity of ER stress activation, and methodological approaches used to evaluate UPR signaling.

Importantly, the current evidence base has several limitations. Many mechanistic insights are derived primarily from cell culture systems and experimental animal models, particularly oxygen–glucose deprivation models and rodent stroke models. However, the complexity of human stroke pathology—including patient heterogeneity, comorbidities, and variations in ischemic duration—may substantially influence therapeutic responses and limit the direct translation of these findings into clinical practice.

From a translational perspective, several practical challenges must also be considered. The therapeutic window for ER stress modulation remains uncertain, as moderate activation of the UPR during the early stages of ischemia may be protective, whereas prolonged activation may promote neuronal apoptosis. In addition, the timing and specificity of therapeutic intervention targeting different UPR branches are likely to be critical determinants of treatment efficacy. Another important consideration is the blood–brain barrier, which limits the delivery of many pharmacological agents targeting ER stress signaling pathways. Furthermore, potential off-target effects and systemic toxicity must be carefully evaluated because ER stress pathways are involved in multiple physiological processes in peripheral organs.

Future research should therefore prioritize several key directions. First, studies should clarify the spatiotemporal regulation of ER stress signaling during different stages of cerebral ischemia and reperfusion. Second, further investigation is needed to define the cell-type–specific regulatory mechanisms of ER stress within the neurovascular unit. Third, greater emphasis should be placed on identifying therapeutic strategies capable of selectively modulating adaptive UPR signaling while minimizing pro-apoptotic responses and systemic toxicity. Finally, integrating advanced experimental models, multi-omics approaches, and clinically relevant translational studies will be essential for bridging the gap between experimental discoveries and effective clinical therapies for ischemic stroke.
